# High rate of species misidentification reduces the taxonomic certainty of European biodiversity databases of ivies (*Hedera* L.)

**DOI:** 10.1038/s41598-024-54735-0

**Published:** 2024-02-28

**Authors:** Marina Coca-de-la-Iglesia, Angélica Gallego-Narbón, Alejandro Alonso, Virginia Valcárcel

**Affiliations:** 1 Departamento de Biología, Universidad Autónoma de Madrid, 28049 Madrid, Spain; 2 TRAGSATEC, Madrid, Spain; 3 Centro de Investigación en Biodiversidad y Cambio Global (CIBC‐UAM), Universidad Autónoma de Madrid, 28049 Madrid, Spain

**Keywords:** Biodiversity, Plant sciences

## Abstract

The digitization of natural history specimens and the popularization of citizen science are creating an unprecedented availability of large amounts of biodiversity data. These biodiversity inventories can be severely affected by species misidentification, a source of taxonomic uncertainty that is rarely acknowledged in biodiversity data management. For these reasons, taxonomists debate the use of online repositories to address biological questions at the species level. *Hedera* L. (ivies) provides an excellent case study as it is well represented in both herbaria and online repositories with thousands of records likely to be affected by high taxonomic uncertainty. We analyze the sources and extent of taxonomic errors in the identification of the European ivy species by reviewing herbarium specimens and find a high misidentification rate (18% on average), which varies between species (maximized in *H. hibernica*: 55%; *H. azorica*: 48%; *H. iberica*: 36%) and regions (maximized in the UK: 38% and Spain: 27%). We find a systematic misidentification of all European ivies with *H. helix* behind the high misidentification rates in herbaria and warn of even higher rates in online records. We compile a spatial database to overcome the large discrepancies we observed in species distributions between online and morphologically reviewed records.

## Introduction

Our knowledge of biodiversity is limited and patchy^1^. Even for one of the most fundamental understandings of biodiversity, species distributions, our knowledge is incomplete and biased^1,2^. However, an increasing number of ecological and phylogenetic studies are demanding from occurrence databases to be able to analyze evolutionary processes and to understand biodiversity patterns^3^. The popularity of citizen science^4^ is constantly providing us with large amounts of biodiversity observations^5^. In parallel, intense global digitization efforts are making natural history collections easily accessible^6,7^. As a result, during the last decades, several online repositories have collected large amounts of spatial information, combining direct field observations and natural history collections (e.g., Global Biodiversity Information Facility-GBIF, https://www.gbif.org/; iNaturalist, https://iNaturalist.org/; SpeciesLink, http://splink.cria.org.br/). However, the extent to which this vast amount of biodiversity data can be used for research purposes ultimately depends on its quality^8^, which is not always guaranteed.

**Figure 1:**
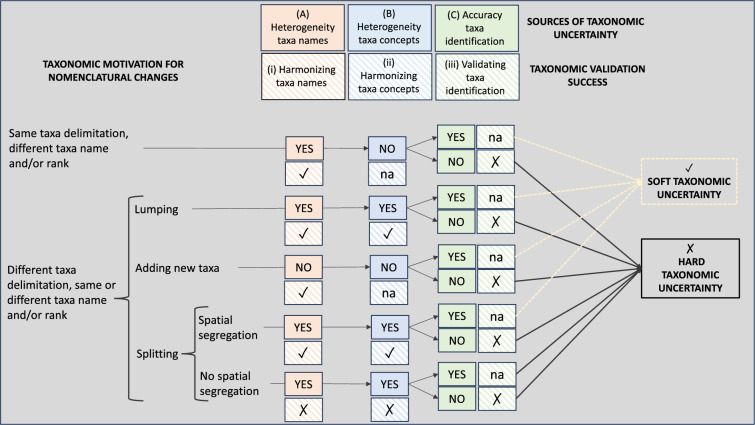
Sources of taxonomic uncertainty in biodiversity databases and the validation process. Biodiversity databases have three main sources of taxonomic uncertainty^12^: (**A**) heterogeneity of taxa names, (**B**) heterogeneity of taxa concepts (different taxa delimitation), and (**C**) accuracy of taxa identification. Heterogeneity in taxa names can be easily solved (soft taxonomic uncertainty) by harmonizing taxa names, if there is no difference in the delimitation of taxa, or also by harmonizing taxa concepts if the differences in the delimitation of taxa are due to merging taxa, the recognition of new taxa, or splitting taxa resulting in spatial segregation. However, if the heterogeneity in taxa names is due to splitting taxa resulting in no spatial segregation (hard taxonomic uncertainty), harmonizing taxa names and concepts cannot always guarantee to solve the issue. Above these two sources of taxonomic uncertainty, if the accuracy of taxa identification is low, a validation of the identification is needed. If the validation requires access to the specimen, then the taxonomic uncertainty of the record is hard regardless of whether according to the other sources of taxonomic uncertainty it was considered soft or hard.

The quality of occurrence databases depends on the extent, precision and representativeness of the geographical records, as well as on the accuracy of identification of these records and their taxonomic coverage^2,8,9^. However, the taxonomic quality standards of biodiversity inventories are heterogeneous and often low when considered together with the geographical quality. For example, even in well-studied regions such as Europe, where large-scale geographical coverage is high^10^, the geographical uncertainty may be high^9^ and the taxonomic quality low depending on the study group^2,11^ and/or the taxonomic sampling unit (species, genera, etc.). Over the last decades, several tools and procedures have been developed to achieve good taxonomic quality in biodiversity inventories^12^. Most of these procedures focus on the importance of harmonizing taxonomic names, which means updating nomenclature by dealing with synonyms and correcting of spelling errors^12^. As a result, there is a plethora of procedures to automatically deal with this first dimension of taxonomic quality in databases^12,13^. However, part of the heterogeneity in taxonomic names is due to the application of different criteria for the delimitation of taxa (different taxonomic concepts) that ends up with different delimitation of taxa (different taxa grouping) because of splitting, merging or adding new taxa (Fig. 1). Dealing with this type of changes may be straightforward when entities are lumped together by synonymizing the name of the taxon that is no longer accepted, or when a new taxon is discovered as no data mining is needed. However, dealing with changes that involve splitting taxa is often a major challenge that is difficult to resolve unless the newly split taxa live in allopatry and geographical filtering can be done. This is underlined by the fact that the information on the taxonomic criterion that is used is often neglected^11,14^, making the harmonization of taxonomic concepts in biodiversity inventories extremely difficult, as it requires in-depth taxonomic knowledge of the group. As a result, the approaches that address this second dimension of the taxonomic quality are scarce. Indeed, only a few studies acknowledge the need to harmonize taxonomic concepts^2,15^. However, a third dimension that is by far the least studied is the assessment of the accuracy of taxa identification, a validation that is in fact rarely even acknowledged in the handling of biodiversity data^2^. This is probably because it is a time-consuming task that often requires access to specimens, which is often impossible or unrealistic due to the intensive labour required^16^. Furthermore, under certain evolutionary scenarios (e.g., short time for speciation, hybridization, or weak reproductive barriers) species identification becomes extremely difficult and its accuracy is not guaranteed^17,18^. Therefore, the enormous value of online repositories as readily available sources of biodiversity data has been debated when addressing questions at the species-level in specific groups of organisms, regions of the world, or geographic or taxonomic scales^3,19^.

**Figure 2:**
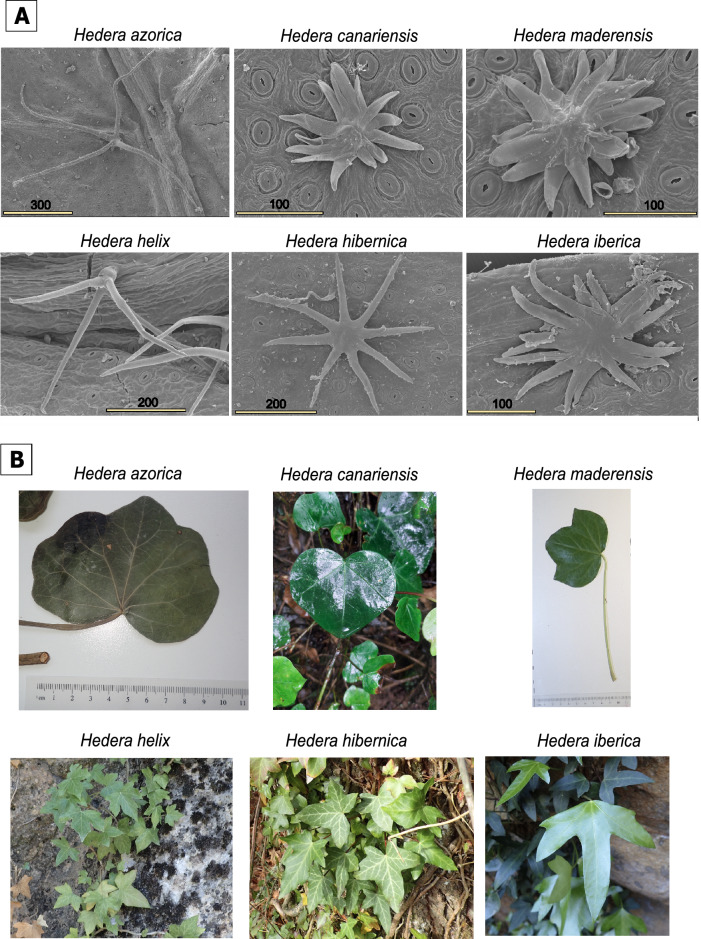
Examples of the typical trichomes and leaves from the vegetative phase in the six *Hedera* species from the west of Europe. (**A**) Types of trichomes. Numbers indicate the scale bar units in micrometres. Scanning microscopic images taken from Valcárcel^33^ representing the typical stellate-multiangulate trichomes of *Hedera azorica* (Portugal, H. Persson, S) and *H. helix* (Ukraine, A.K. Skvortsov, M0080111), stellate-rotate trichomes of *H. hibernica* (Spain, C. García González, 52385JACA) and the typical scale-like trichomes of *H. canariensis* (Spain, V. Vasak, BR-SP852826), *H. maderensis* (Portugal, M. Velayos, MA655340) and *H. iberica* (Spain, V. Valcárcel 391VV01(11), MAUAM). (**B**) Types of leaves from vegetative branches.

This is the case for ivies (*Hedera* L.), a small genus with recent diversification^20^ and extensive hybridization^21^, where species delimitation has long been controversial^22^ and species identification difficult^23^. Identification of ivy species is based on inconspicuous microscopic characters (trichomes) that are difficult to interpret while macroscopic characters (leaves, flowers or fruits) tend to be of little taxonomic importance^22^. In fact, the first feature in the diagnosis of ivy species is the identification of the type of trichomes, while leaves are considered to be secondary diagnostic characters or even useless^22^ (Fig. 2). Even in cases where leaves are used for species diagnosis, it is the combination of leaf and trichome characteristics that allows species identification. As the identification of ivy species ultimately requires distinguishing the robust but subtle differences in their microscopic trichomes that is challenging even for professional botanists^23^, we anticipate a high taxonomic uncertainty in biodiversity inventories whether they come from natural history collections or citizen science observations. We also expect that this limitation will be particularly severe in those regions of Europe where several ivy species live in close contact. This is the case of the Iberian Peninsula (mainland Portugal and mainland Spain), the only region in Europe where three ivy species occur and share their range boundaries^24^.

In this study we aim to assess the type and rate of taxonomic errors in the natural history collections of the European *Hedera* species in order to validate the utility of online records in accurately reflecting the species distributions. Ultimately, we aim to produce an occurrence database with high taxonomic and geographic quality standards. To this end, we first compiled a database of records from morphologically reviewed herbarium specimens to assess the patterns (type and rate) of the taxonomic errors. We then used this database to identify European regions with low taxonomic uncertainty and extracted the records from these European regions from curated online databases. Finally, we merged the morphologically reviewed database with the online curated records to provide a georeferenced database with high taxonomic and geographic quality standards for *Hedera*.

## Material and methods

### Study species

We analyze six of the twelve species of *Hedera* (*H. azorica*, *H. canariensis*, *H. helix*, *H. hibernica*, *H. iberica*, and *H. maderensis*). *Hedera* is a genus of lianas native to the Old World, where it occurs throughout North Africa and Eurasia from the Azores to Japan. We followed the taxonomic criterion of Valcárcel and Vargas^22^, which is based on that of McAllister and Rutherford (see references in Valcárcel and Vargas^22^), except for the recognition of the Iberian ivy as a species (*H. iberica*)^25^ and not as a subspecies of the Madeiran ivy (*H. maderensis* subsp. *iberica*). According to this criterion, there are 12 species (14 taxa) in *Hedera*, seven of which are native to Europe (*H. azorica*, *H. canariensis*, *H. helix*, *H. hibernica*, *H. iberica*, *H. maderensis* and *H. pastuchovii* subsp. *cypria*) and six of which converge in western Europe and are our study species (all except *H. pastuchovii* subsp. *cypria*, which is an endemic restricted to southwestern Cyprus).

### Study area

**Figure 3:**
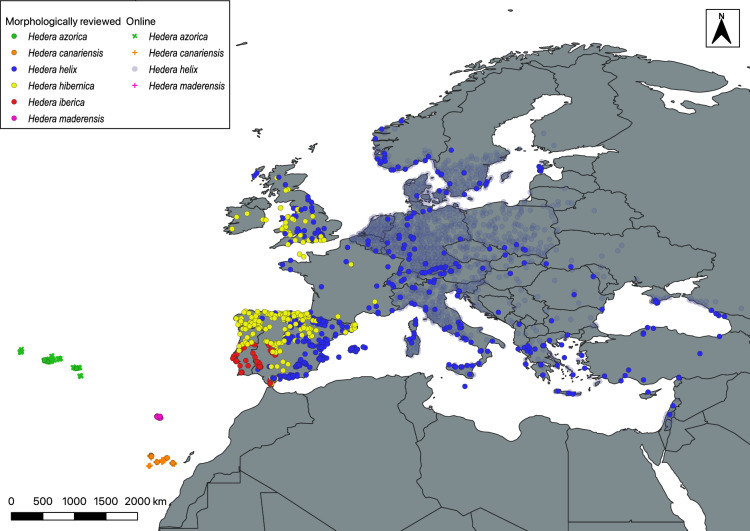
Point-occurrence map of the six European species of *Hedera* native in the west of Europe with taxonomic and geographic coverage (MixOcc database) generated in QGIS version 3.4.3-Madeira^45^. This includes morphologically reviewed records (TaxRev database) for all species (Solid circles), as well as online records (those not morphologically reviewed) for all species (light circles and crosses) except *H. hibernica* and *H. iberica*. The exclusion of the latter two species is due to their entire distribution being within regions characterized by high taxonomic uncertainty—i.e., regions with more than one ivy species sharing ranges.

Our study area is southwestern Europe, which includes the Iberian Peninsula (mainland Spain and mainland Portugal) and the Macaronesian archipelagos of Madeira, the Azores and the Canary Islands. This region harbors half of the species of *Hedera*, and it is the main diversity center of the genus as inferred from morphological, ploidal and genetic patterns of variation^26^. Three of the species native to southwestern European species are endemic to the three Macaronesian archipelagos (*H. azorica* in the Azores*, H. canariensis* in the Canary Islands*, H. maderensis* in Madeira; Fig. 3). The remaining three species occur in southwestern of mainland Europe (Fig. 3), with *H. iberica* as a local endemic restricted to southwestern Iberian Peninsula, *H. hibernica* as a widespread species occurring mainly on the Atlantic side of western Europe (from the southwestern Iberian Peninsula to France, the UK and Ireland), and *H. helix* as the most widespread species occurring throughout Europe*.* The three mainland species come into contact in the Iberian Peninsula, with *H. helix* contacting *H. iberica* in the southwest and *H. hibernica* all the way from the north to the south across the western limit of its range. Similarly, *H. hibernica* contacts with *H. iberica* in the south of its range in Portugal. Outside the Iberian Peninsula, *H. helix* contacts with *H. hibernica* throughout the latter’s range, which includes France, the UK and Ireland, and occurs as the sole ivy species in the rest of its range. Because of this pattern of species contact and the difficulty of species identification^24^ (see below), we expect the greatest level of taxonomic uncertainty in the identification of European ivy species to occur in western Europe, and particularly in the southwest (Iberian Peninsula).

### Habitat

The species studied generally grow in shady, moist places such as riverbanks, ravines or forest understories, with varying degrees of tolerance to dry, sunny locations such as exposed rock faces^20,27^. Indeed, some species, such as the common ivy (*H. helix* L.), can survive over a wide range of temperatures and rainfall regimes^27^. In contrast, some other species are restricted to extremely humid and shady understories such as *H. maderensis*, *H. canariensis* or *H. azorica*, which occur in the typical Macaronesian subtropical laurel forests^20^; or *H. iberica*, which also has a very strict habitat affinity for warm humid sites in the southwest of the Iberian Peninsula^27^. In terms of substrate, ivies grow on almost any type of soil, except those that are extremely acidic, very wet or waterlogged^28^. In general, they seem to prefer well developed, rich, alkaline soils and rocks^28^. However, some species, such as *H. hibernica*, may prefer more acidic soils, as suggested by its distribution range in the Iberian Peninsula^27^. Ivies constitute a relevant element of the riparian and forest vegetation in Europe. They are often the sole representative of the liana element^29^, and they are even key species for specific vegetation types (*H. canariensis* is one of the key species of the Canarian laurisiva^30^). Furthermore, *H. helix* has been proposed as an indicator of forest habitat quality in the Central European highlands^31^.

### Species identification of west European ivies

The most important diagnostic character in *Hedera* is the type of trichomes (scale-like, stellate-rotate and stellate-multiangulate^32^; Fig. 2A), which is the first step when identifying European ivy species^22^. Globally, there are only two ivy species with stellate-multiangulate trichomes and both are endemic to the west of Europe (*H. azorica* and *H. helix*; Fig. 2A). There is also only one representative of the stellate-rotate type that is also endemic to western Europe (*H. hibernica*; Fig. 2A), whereas the remaining nine *Hedera* species have scale-like trichomes including the rest of our studied species (*H. canariensis, H. iberica* and *H. maderensis*; Fig. 2A). Indeed, the only area in the world where the three types of trichomes converge is the Iberian Peninsula in Europe^24^, with *H. helix* representing the multiangulate type, *H. hibernica* as the representative of the rotate type and *H. iberica* representing the scale-like type. Although the trichomes of the three species are quite distinct, it is common to observe individuals with intermediate features in the regions where they contact. Indeed, throughout the distribution of *H. hibernica* (from the UK to Spain), whenever it comes into contact with *H. helix*, there are populations whose individuals display intermediate forms of trichomes between the typical multiangulate of *H. helix* and the typical rotate of *H. hibernica*^24^.

In the case of intermediate trichome forms between *H. hibernica* and *H. iberica*, the vegetative leaves may help to identify the species, as *H. iberica* typically has characteristic deeply lobate leaves that have not been seen in *H. hibernica* (Fig. 2B)*.* However, the two species overlap in the range of variation in leaf morphology and, therefore the species identification often ends up relying on trichomes^22^. In the case of the intermediate trichomes between *H. helix* and *H. hibernica*, leaves (or any other macromorphological trait so far analyzed) do not help in species identification, as the two species show high variation and overlap^22^ (Fig. 2B).

Finally, within the three Macaronesian ivy species, *H. azorica* is quite distinct because of its multiangulate trichomes (Fig. 2A), which strongly contrast with the typical scale-like trichomes of *H. canariensis* and *H. maderensis* (Fig. 2A). In fact, the trichomes of *H. azorica* could only be confused with those typical of the mainland species *H. helix* (Fig. 2A), from which it can be distinguished by the degree of lobulation of the leaves of the vegetative branches (Fig. 2B) and the size and shape of the leaves of the reproductive branches^22^. The distinction between *H. maderensis* and *H. canariensis* (both displaying similar scale-like trichomes; Fig. 2A) is entirely based on the leaves of the vegetative branches, which are typically entire and heart-shaped in *H. canariensis* (Fig. 2B) and lobate with three wide lobes in *H. maderensis*^22^ (Fig. 2B).

### Compilation of the morphologically reviewed database (TaxRev)

To compile an occurrence database with high certainty on species identification (hereafter “TaxRev database”), we used the specimens studied in Valcárcel’s Ph.D. thesis, partially published^22,23,33,34^. Only native records of the specimens of the six *Hedera* species from western Europe were selected, representing the entire native range of the six species. These specimens originally came from 40 herbaria (Table S1) and from field collections of V. Valcárcel and P. Vargas teams in Austria, England, France, Germany, Greece, Ireland, Italy, and Scotland, deposited in MA and MAUAM (see TaxRev for vouchers and herbarium codes). The records are identified in the database as “Herbarium loan & Valcárcel PhD collection”. Additionally, we analyzed 479 individuals from 117 populations of the four native ivy species collected in the Iberian Peninsula and Madeira (identified in the database as “NiDEvA project”). These individuals were originally collected by the authors of this study during the fieldwork of a scientific project (NiDEvA project, CGL2017-87198-P, Spanish Ministry of Economy, Industry and Competitiveness), the specimens were deposited in the herbarium of the *Universidad Autónoma de Madrid* (MAUAM, see TaxRev for vouchers and herbarium codes) and were partially used in two molecular studies^20,27^.

Species identification was carried out by V. Valcárcel following the taxonomic criterion of Valcárcel and Vargas^22^ and was mainly based on micromorphological information from foliar trichomes and macromorphological characters, mostly from vegetative leaves. All the information recorded was homogenized. In the case of locality and habitat, the information was retained both as originally recorded and in its revised versions, including typographical corrections and English translation. The TaxRev database is available in Zenodo^35^.

### Evaluation of the taxonomic uncertainty of TaxRev

We assessed the extent of errors in the identification of ivy species in the TaxRev database. To do this, we analysed the number of records in which the original identification of the taxa changed after the revision by the taxonomist of the genus, V. Valcárcel. To avoid problems of circularity, we first removed all records originally identified by V. Valcárcel and other botanists involved in the taxonomic proposal we followed, including H. A. McAllister, P. Vargas, and A. Rutherford. The resulting records were first coded as correct or incorrect, whether there was no change between the name of the original identification and the name after the morphological revision, or the species name was changed. We then subdivided the incorrect records (different names) into four categories to reflect the different types of taxonomic uncertainty and the procedures of taxonomic validation (Fig. 1). “Hard taxonomic changes” identify name changes associated with taxa splitting resulting in no spatial segregation or spatial segregation with nomenclatural confusion, as all these cases reflect taxonomic changes in taxa concepts that cannot be harmonized by standard nomenclatural validation procedures (Supplementary Note 1). “Soft taxonomic changes” identify name changes that do not imply changes in the delimitation of the taxa or those that imply either the merging of taxa or the splitting of taxa resulting in allopatric taxa, as in all these cases the taxonomic changes can be harmonized through standard nomenclatural validation procedures (Supplementary Note 1). Finally, for those records where the differences between the original and the revised identification are due to a traceable misidentification, which may or may not be associated with a nomenclatural change (Supplementary Note 1), we identified them as “Misidentifications”, and those without original identification at the species level as “Not identified”.

We then performed Generalized Linear Models (GLM) to assess whether the species identification error (categorical response variable with two levels: YES/NO) varied according to the species and/or the expected geographic taxonomic uncertainty. For the species effect, we used a six-level categorical variable corresponding to the six recognized species. For the geographical expected taxonomic uncertainty, we used a numerical variable indicating the number of species sharing range boundaries in the region of origin of the record (3 spp. in mainland Spain; 2 in mainland Portugal, France, United Kingdom, and Ireland; 1 in the rest of the countries and the Macaronesian islands). When there was only one ivy species, we considered the regions (countries or isolated areas) to be of low expected taxonomic uncertainty, while the regions with more than one ivy species were considered as with high taxonomic uncertainty.

We used one-dimensional, two-level contingency table tests with chi-squared goodness of fit to test the hypothesis that correct and incorrect identifications are equally frequent. These chi-squared tests were performed on the entire dataset, per species, per region of taxonomic uncertainty (low vs. high), and within each of the regions of expected high taxonomic uncertainty (France, UK, Ireland, mainland Portugal and mainland Spain). Finally, to assess the patterns of identification errors per species, we built a classification matrix. In this matrix, the six columns indicate the species according to V. Valcárcel revision and the rows summarize the original taxa identifications of herbarium specimens. All these analyses were performed in R (R Core Team 2023) using the following packages: tibble^36^, tidyverse^37^, dplyr^38^, and countrycode^39^.

### Compilation of the spatial point database (MixOcc)

In order to provide a point occurrence database with taxonomic certainty, we first compiled a spatial point database with records from the morphologically reviewed TaxRev database. This includes all records for which spatial coordinate information was originally provided (Supplementary Note 2). In addition, the records where spatial coordinate information was not available but had detailed locality descriptions were georeferenced using GeoLocate Web Application^40^ and Google Maps (Supplementary Note 2). Whenever more than one option was found, or GeoLocate failed to find a location, we used GoogleMaps to select the most likely site (Supplementary Note 2), i.e. the one with the highest probability of the species occurrence (forested, riverine or rocky sites). Coordinates and spatial uncertainty data are provided following the Darwin Core Standard^41^ (Supplementary Note 2).

To avoid the undesirable effects of low geographical coverage, we decided to extract a selection of records from two curated online databases of *Hedera* for the geographical gaps detected in the occurrences obtained from TaxRev (see below). However, we could not assess the accuracy of species identification of these online records, which is a serious limitation due to the difficulties of ivy species identification (see above). Therefore, we decided to include only the online records from the regions in Europe with low expected taxonomic uncertainty, that is, from central and eastern European countries and from the three Macaronesian archipelagos (Azores, Madeira and Canary Islands). We used this approach because the expected taxonomic uncertainty in these regions is low, and most of the taxonomic uncertainty is due to the taxonomic criterion applied and outdated nomenclature. Given that these two sources of taxonomic uncertainty can be easily addressed with a taxonomic validity test that does not require access to the specimens, we ensure the taxonomic quality standard of the database. To extract online records from central and eastern continental Europe, we filtered a curated database^42^ to retain European *Hedera* records from countries with only one native ivy species. The original source for all these records was the Global Biodiversity Information Facility (GBIF; www.gbif.org, see DOI references for original downloads in Supplementary Note 2). To extract online records from the Macaronesian archipelagos, we used another curated database^20^. The original sources for these Macaronesian records were the Azorean Biodiversity Portal^43^ and the Biodiversity Data Bank of the Canary Islands (www.biodiversidadcanarias.es). All the online databases had already been curated by removing cultivars, duplicate localities, and records with low spatial quality (i.e., low precision or erroneous coordinates). In addition, a distance buffer was already applied in the two databases to minimize the impact of unequal sampling effort (10 km for continental areas^44^ and 1 km for Macaronesian archipelagos^20^). A taxonomic validation was carried out in the two databases to unify the taxonomic criterion according to Valcárcel and Vargas^22^, update the nomenclature and correct typographical errors. Finally, we merged the filtered records from these online databases together with the georeferenced records from TaxRev and compiled the spatial point database (“MixOcc” database; available in Zenodo^35^).

The MixOcc database was used to build point-occurrence maps with QGIS version 3.4.3-Madeira ^45^, all of them available in Zenodo^35^.

## Results

### Taxonomic and geographic coverage and quality of TaxRev and MixOcc Databases

We present a database of high taxonomic quality (TaxRev) containing 1280 records (localities/populations) of six European ivy species. This database was compiled from a total of 2276 morphologically reviewed individuals and represents all European ivy species except for *H. pastuvovii* subsp. *cypria*, a local endemic from the southwest of the island of Cyprus. We obtained spatial points for 880 of these records with spatial information for four species (*H. helix*, *H. hibernica*, *H. iberica* and *H. maderensis*; Table S2, Fig. 3), although the geographical coverage of *H. helix* and *H. hibernica* was low in some regions (Table S3; see solid circles in C and E Europe and France and Ireland in W Europe in Fig. 3). For the remaining two species (*H. azorica*, *H. canariensis*), the spatial points obtained from TaxRev did not fully represent their entire range (Table S2, see circles in Fig. 3). By using online records from regions with low taxonomic uncertainty for ivies (regions with one ivy species) we improved the geographic coverage for *H. azorica* and *H. canariensis* across their entire distributions, as well as for *H. helix* in C and E Europe (Table S2, see blue light circles and crosses in Fig. 3). As a result, the MixOcc database contains 3252 point occurrences with high geographical coverage (Tables S2, S3, Fig. 3).

### Geographical and taxonomic patterns of errors in species identification in the morphologically reviewed database

The results of the GLM showed that species identification errors in the TaxRev database varied with the number of ivy species sharing range boundaries (1, 2 or 3) per region (country or island), and with the species (Table S4). *Hedera hibernica* and *H. iberica* have a positive effect on species identification errors (i.e., more incorrectly identified records), while *H. helix* has a negative effect (i.e., more correctly identified records) and the remaining three species do not have any effect (Table S4). The number of ivy species sharing range boundaries per region also has a positive effect on the species identification errors, i.e., the more ivy species in close proximity per region, the more species identification errors (Table S4).

Overall, the percentage of incorrect identifications in the TaxRev was 47%, and we could not reject a random effect on the correct vs. incorrect species identifications (538 vs. 473; Table 1). Incorrect identifications were also significantly more frequent than correct identifications within regions with more than one ivy species in close proximity (213 correct vs. 343 incorrect, Table 1). On the contrary, incorrect identifications were significantly less frequent than correct identifications within regions with only one ivy species (321 correct vs. 128 incorrect, Table 1). When analyzing the frequency of errors within each of the five regions with high taxonomic uncertainty (i.e. more than one ivy species sharing range boundaries), we found that incorrect identifications were significantly more frequent than correct identifications in the Iberian Peninsula (mainland Portugal and mainland Spain) and we could not reject a chance effect in the case of the UK. The small number of observations in France and Ireland prevented the identification of a pattern (Table S5).

**Table 1:** Error patterns of *Hedera* species identification (types and rates in %) obtained from TaxRev without records originally identified by *Hedera* taxonomists. This information is provided for the whole dataset (N = 1011 records), per geographical region according to the expected taxonomic uncertainty (“Low” in regions with only one ivy species vs. “High” in regions with 2 or 3 spp. sharing range boundaries; N = 1005) and per species. Significant p-values from Chi-squared test are indicated as: ****p ≤ 0.0001, ***p ≤ 0.001, **p ≤ 0.01, *p ≤ 0.05, ", whereas p ≤ 0.08 are indicated as m.s. (marginal signficant) and p > 0.08 as n.s. (not significant). Mis: incorrect identifications attributed to misidentifications; NoIden: lack of original identification at the species level. Hard: incorrect identifications attributed to hard taxonomic changes (changes that cannot be validated with standard procedures of taxonomic validations). Soft: incorrect identifications attributed to soft taxonomic changes (changes that can be validated with standard procedures of taxonomic validations).

	N	Correct:Incorrect identifications	Correct identifications	Incorrect identifications				
				Total	Mis	NoIden	Hard	Soft
Total	1011	538:473*	538	473	180	190	26	77
			53%	47%	18%	19%	3%	8%
Regions with low uncertainty	449	321:128****	321	128	30	45	9	44
			71%	29%	7%	10%	2%	10%
Regions with high uncertainty	556	213:343****	213	343	149	144	17	33
			38%	62%	27%	26%	3%	6%
*H. azorica*	53	19:34^m.s^	14	26	19	6	0	1
			35%	65%	48%	15%	0%	3%
*H. canariensis*	53	19:34*	19	34	4	9	0	21
			36%	64%	8%	17%	0%	40%
*H. helix*	619	475:144****	475	144	12	82	0	50
			77%	23%	2%	13%	0%	8%
*H. hibernica*	222	24:198****	24	198	121	70	4	3
			11%	89%	55%	32%	2%	1%
*H. iberica*	55	1:54****	1	54	20	20	13	1
			2%	98%	36%	36%	24%	2%
*H. maderensis*	22	5:17**	5	17	4	3	9	1
			23%	77%	18%	14%	41%	5%

When analyzing the frequency of errors per species, incorrectly identified records were significantly more frequent than correctly identified records for all species, except for *H. helix* for which correctly identified records were significantly more frequent than incorrect records (Table 1). The type of error varied between species with a constant pattern of species misidentification with *H. helix* and *H. canariensis* (Table 2). Most of the incorrect identifications in *H. hibernica* are due to original misidentifications with *H. helix* (114 out of the 198 incorrect records). The incorrect identifications in the *H. iberica* records have different causes, as 20 of them are due to a misidentification (19 with *H. helix* and 1 with *H. hibernica*; Table 2 ), 13 is due to a soft taxonomic change (with *H. maderensis* var./subsp. iberica), 13 to a hard taxonomic change (with *H. canariensis* and *H. helix* subsp./var. *canariensis*) and 20 are due to the lack of any original identification at the species level. The most common error in the species identification of *H. azorica* and *H. maderensis* records is to assign them to *H. canariensis*, while the errors in *H. canariensis* records are mostly due to the lack of any original identification at the species level or to soft taxonomic changes (with *H. helix* var./subsp. *canariensis*; Table 2).

**Table 2:** Classification table obtained from TaxRev without records originally identified by *Hedera* taxonomists (N = 1011 records). In bold we identified the taxa recognized in Valcárcel and Vargas^22^.

	Morphologically-reviewed species identification					
	*H. azorica*	*H. canariensis*	*H. helix*	*H. hibernica*	*H. iberica*	*H. maderensis*
Original identification						
*** H. algeriensis***	0	0	**1**	0	0	0
* H. arborea*	0	0	**1**	0	0	0
*** H. azorica***	**14**	0	0	0	0	0
*** H. canariensis***	**12**	**19**	0	**2**	**6**	**6**
* H. caucasica*	0	0	**1**	0	0	0
* H. colchica*	0	0	**2**	0	0	0
* H. congesta*	0	0	0	**1**	0	0
*** H. helix***	**3**	**4**	**475**	**103**	**15**	**3**
H. helix var./subsp./form						
* arborea*	0	0	**1**	0	0	0
* azorica*	**1**	0	0	0	0	0
* borealis*	0	0	0	**1**	0	0
* burgalensis*	0	0	**1**	0	0	0
* canariensis*	**2**	**21**	0	**2**	**7**	**3**
* digitata*	0	0	0	**1**	0	0
* floribunda*	0	0	**2**	0	0	0
* helix*	0	0	**26**	**11**	**3**	**1**
* hibernica*	**2**	0	0	**3**	0	0
* minima*	0	0	**1**	0	0	0
* paniculata*	0	0	**1**	0	0	0
* pedunculata*	0	0	**1**	0	0	0
* poetarum*	0	0	**2**	0	0	0
* rhizomatifera*	0	0	**3**	0	**1**	0
* sarniensis*	0	0	**1**	**3**	0	0
* sarracena*	0	0	0	**1**	0	0
* typica*	0	0	**1**	0	0	0
*** H. hibernica***	0	0	**9**	**24**	**1**	0
*** H. iberica***	0	0	0	0	**1**	0
*** H. maderensis***	0	0	0	0	0	**5**
* H. maderensis var./subsp.*						
* iberica*	0	0	0	0	**1**	0
* maderensis*	0	0	0	0	0	**1**
*H. poetarum*	0	0	**4**	0	0	0
*H. taurica*	0	0	**4**	0	0	0
Not identified	**6**	**9**	**82**	**70**	**20**	**3**

## Discussion

The taxonomic quality of biodiversity inventories depends on the accuracy of taxa identification, which ultimately depends on the knowledge and expertise of the identifier^46–48^ and the resources available for the identification. The expertise of the identifier is particularly important for groups of organisms with high taxonomic controversy and difficult species identification, such as *Hedera*. Indeed, we found an average error rate of 47% in the original species identification of herbarium specimens (Table 1). Most of these errors are due to an insufficient knowledge of identifiers about ivies’ diagnostic characters resulting in the absence of an original species identification or in misidentifications (19% and 18%, respectively, Table 1), and are concentrated in the regions with more than one ivy species.

Current general knowledge on species misidentification rates is limited in the scientific literature, but the studies carried out indicate a high variability between species and groups of organisms (0–56% for freshwater mussels^49^; 0.6–41.1% for European ungulates^47^; 2.3–5.3% for French plant species^48^). The high misidentification rates we observed for some of the European ivy species (55*%* for *H. hibernica*, 48% for *H. azorica* or 36% for *H. iberica*, Table 1) are not surprising, given that even experienced botanists can face challenges in identifying ivies due to the difficulty in interpreting leaf trichomes^23^. Trichomes are small and easily lost during the development of individuals, thus, the first limiting factors for accurate identification of ivies are having an adequate magnifying glass in terms of quality and degree of magnification, and the plant material used. In fact, half of the misidentified specimens consisted of herbarium sheets containing only reproductive branches (see TaxRev database), a growth phase that rarely retains trichomes on its leaves. However, if these methodological problems were the only obstacle to accurate identification of ivies, the misidentification rate would be comparable for all species, whereas our misidentification rates vary widely between species.

The species-dependent pattern of misidentification rates in ivies is explained by both intrinsic biological causes and human-based cascade errors. First, the two main speciation mechanisms in *Hedera* (allopolyploidy^50^ and geographic isolation with slight niche shifts^20,27^) have resulted in complex patterns of species variation in trichomes and leaves^22^. For example, the stellate-rotate trichomes, found only in *H. hibernica*, show intermediate features between the other two types of trichomes in *Hedera* (stellate-multiangulate and scale-like, Fig. 2A ^22^). These intermediate features are interpreted as the morphological footprint of the hybrid origin of the tetraploid *H. hibernica* from two diploid ancestors, one with stellate-multiangulate trichomes (like *H. helix*) and the other one with scale-like trichomes^50,51^. In addition, the scale-like trichomes of *H. iberica* show certain characteristics that are more typical of the stellate-rotate trichomes of *H. hibernica* (small central part and rays of very regular length) than those of the typical scale-like trichomes of the other *Hedera* species, which have large central parts and rays of more irregular length^22^. This morphological similarity between the trichomes of *H. iberica* and those of *H. hibernica* may also reflect the evolutionary history of the species; a recent study has suggested a pattern of nested speciation of *H. iberica* within *H. hibernica*^27^. Interpreting the trichomes of these species can therefore be very difficult. In the case of *H. iberica* because they can be confused with those of *H. hibernica*, and, in the case of *H. hibernica*, because they can be confused with those of either *H. iberica* or *H. helix*^24^. However, misidentifications between *H. hibernica* and *H. iberica* are rarely recorded in our database. Instead, the most common misidentification of the two species is with *H. helix* (Table 2). These results suggest that the high misidentification rates we observe are not exclusively a direct consequence of the difficulty in ivy species identification.

Consistent patterns of error in species identification (systematic misidentifications) often occur between morphologically similar species, such as *H. hibernica* and *H. helix*. However, systematic misidentifications are less likely to occur between morphologically distinct species unless they live in sympatry, which is not the case for *H. iberica* and *H. helix*. Systematic misidentifications have a significant impact on the propagation of errors^52^, which may ultimately have an amplifying effect on the original misidentification and a collateral effect of spreading the misidentification to other species (cascade errors^14^). We interpret that the persistent confusion of *H. hibernica* with *H. helix*, originally caused by their morphological resemblance, has contributed significantly to the spread of the idea that all ivies in mainland Europe are the common ivy. A feedback process that has, in turn, contributed to increasing the effect of the misidentification of *H. hibernica* with *H. helix*, while propagating the confusion to the quite distinct *H. iberica*. Finally, we wonder whether the high rate of correct species identification we obtained for *H. helix* (77%, Table 1) is the result of a greater knowledge of this species, or of a mere chance effect due to the widespread misconception that the common ivy is the only *Hedera* species in Europe (excluding the Macaronesian archipelagos).

Another interesting case of systematic error is the frequent misidentification of *H. azorica* with the quite distinct *H. canariensis.* In this case we attribute the systematic confusion to a cascade error originating from a long-standing nomenclatural confusion over the epithet “canariensis”. During the nineteenth and twentieth centuries, several authors used “canariensis” to refer not only to the ivies from the Canary Islands, but also to those from Madeira, the Azores, and even to those from the southwestern Iberian Peninsula with trichomes other than stellate-multiangulate, which we now recognize as *H. iberica* and *H. hibernica*^33^. This is probably also the reason why *H. canariensis* is the second species with which most western European ivies are confused (after *H. helix*, Table 2), although it is the most morphologically distinct *Hedera* species in western Europe because of its entire heart-shaped vegetative leaves.

The systematic misidentifications of all European ivies with *H. helix* and *H. canariensis* that we observed provide an explanation for the highly discordant results obtained when comparing the distribution ranges of morphologically reviewed and online records (Figs. 3 vs. 4). For example, the wide distribution of *H. helix* in the Iberian Peninsula according to online records and the contrasting narrow distribution of *H. hibernica* (Figs. 3 vs. 4) are probably an amplifying effect of the systematic confusion of ivies with *H. helix* during field identifications. Unfortunately, the few taxonomic validation systems that have been proposed to deal with species misidentification in biodiversity databases^53,54^ are unlikely to solve the issues with ivies. The use of machine learning for species identification^55^ can be a powerful tool to validate inaccurate identifications from online photographic records^53,54^. However, this method may not work for *Hedera* as the most common features presented in photographic records are macromorphological characters of limited diagnostic use in European ivies (leaves, flowers or fruits; see *Hedera* records in GBIF or iNaturalist). Similarly, the use of predictive niche modelling, which has proved to be highly effective in correcting inaccurate identifications in online records^19^, may not yield satisfactory results for European ivies either, as their niches overlap considerably, especially those of the most commonly confused species^20,27^.

**Figure 4:**
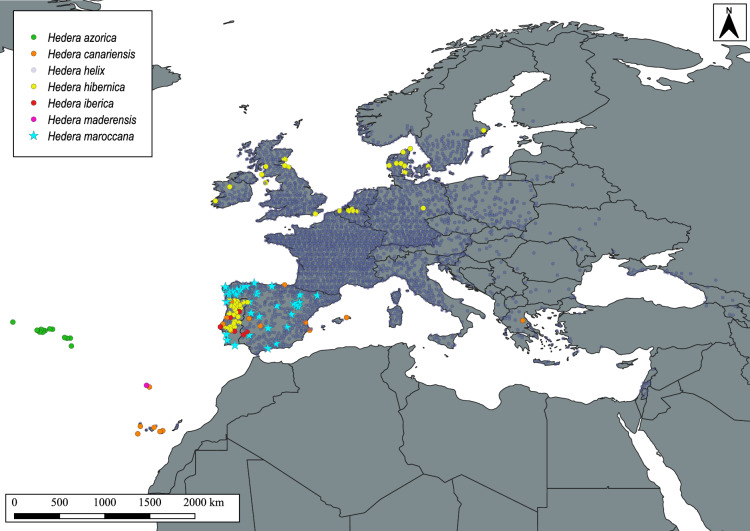
Point-occurrence map of the European species of *Hedera* occurring in the west of Europe according to the online databases analysed (GBIF, Azorean Biodiversity Portal, Biodiversity Data Bank of the Canary Islands) after a careful geographic curation of the records and the harmonization of taxa names and concepts according to the taxonomic criterion used in Valcárcel and Vargas^22^. Generated in QGIS version 3.4.3-Madeira^45^.

Another notable disagreement between online and morphologically reviewed records is the occurrence of *H. canariensis* and *H. maroccana* in the Iberian Peninsula, which are not native to this area. While this can be easily resolved by filtering with native ranges, the application of this curation step requires in-depth knowledge of *Hedera* to discard a naturalized origin. The naturalization of *H. canariensis* in the geographical areas indicated (Fig. 4) is unlikely because the climate in these areas is not as warm and humid as that preferred by *H. canariensis*^20^. The case of *H. maroccana* is different, as the climate that this species occupies in its native range in Morocco is also present in part of the areas in mainland Europe where it has been recorded^20^ (Fig. 4). However, we can also rule out a naturalized origin for *H. maroccana*, as this species has rarely been found to be naturalized^24^, although it is often used as an ornamental plant in gardens on the Iberian Peninsula^24^ and in fences in southern Europe^23^. Therefore, the most likely explanation for the European field observations of *H. canariensis* and *H. maroccana* is the misidentification of the individuals, most likely due to cascade errors (as described above).

The high rates of species misidentification in the European ivies may have unknown consequences, extending the cascade effects beyond taxonomy^14^. Indeed, ivies, and in particular *H. helix*, have been used as model plants for the analyses of vegetation changes^29^, monitoring of climate change^56,57^, medicinal uses^58,59^ and industrial applications^60^. The conclusions drawn from this type of studies ultimately depend on a precise knowledge of the morphological and geographical delimitation of the species. However, our knowledge of the distribution of ivy species is poor, despite the large amount of geographic information available on European ivies^61^. On the one hand, the morphologically reviewed database we compiled has significant spatial gaps in certain regions of Europe (Canary Islands, the Azores, France, C and E mainland Europe, and to a lesser extent in Great Britain; Table S3, Fig. 3). As spatial gaps have undesirable effects on other dimensions of species knowledge^62^, the use of high quality taxonomic database for fine-scale analyses is limited for four of the six European ivy species (*H. azorica*, *H. canariensis*, *H. helix* and *H. hibernica*). On the other hand, we have evidence that the abundant online records that could help to fill these spatial gaps have large taxonomic uncertainty that cannot be solved by taxonomic validation procedures, thus limiting their use to the regions with low expected taxonomic uncertainty. The MixOcc database compiled here, provides a good balance between taxonomic certainty and geographic coverage. However, there are persistent geographical gaps that need to be properly addressed, particularly in France and Ireland, and to a lesser extent in the UK and several eastern European countries (Fig. 4). This means that despite the perception that biodiversity inventories are no longer needed, especially in developed and extensively studied parts of the world such as Europe^2,10,63^, field inventories are still needed, even for highly conspicuous plants such as ivies.

To advance on the challenges that lie ahead of the biodiversity inventory of *Hedera* in Europe, we propose to combine scientific botanical collections of vegetative branches, which will increase the representation of ivies in European herbaria, with the improvement of citizen science procedures for *Hedera* observations. When collecting and identifying *Hedera* species, we encourage (1) the use of a 10× (preferably 20×) magnifying glass to examine the microscopic but essential features for ivy species diagnosis (trichomes), and (2) always include photographic records. The photographic records must include good pictures of (2a) the trichomes (taken between veins on the underside of a vegetative leaf and using the magnifying glass), (2b) the general aspect of the vegetative branches so that we can examine the phenotypic variation of the leaves, and (2c) a detail of a vegetative leaf representing the shape that predominates in the individual.

### Supplementary Information

Supplementary Information.

Supplementary Tables.

## Data Availability

The original specimens used in this study are deposited in 40 herbaria listed in Supplementary Table 1. Each record in the TaxRev database is linked to the herbarium where it is deposited (see field “HB” in the TaxRev database) and to the deposit number of the specimen, if any (see field “NumHB” in the TaxRev database). Loan forms are available at the hosting herbaria (MA herbarium of the *Real Jardín Botánico de Madrid*, and UPOS, herbarium of the *Universidad Pablo de Olavide*). The specimens sampled in the field are deposited in two herbaria (MA herbarium of the *Real Jardín Botánico de Madrid*, and MAUAM herbarium of the *Universidad Autónoma de Madrid*), deposit number is provided, when available (see field “NumHB” in the TaxRev database). The authors declare that sampling permits were not required. The databases (TaxRev and MixOcc) and the point-occurrence maps used in this paper are deposited at Zenodo repository: https://doi.org/10.5281/zenodo.8138495.
