# Supplementary material for: High rate of species misidentification reduces the taxonomic certainty of European biodiversity databases of ivies (Hedera L.)
Source: Sci Rep. 2024 Feb 28;14:4876. doi: 10.1038/s41598-024-54735-0 (PMC10902322; doi:10.1038/s41598-024-54735-0)
Supplement: Supplementary file 1 — Supplementary Information. [file 41598_2024_54735_MOESM1_ESM.docx]

**High rate of species misidentification reduces the taxonomic certainty of European biodiversity databases of ivies (*Hedera* L.)**

Marina Coca-de-la-Iglesia^1,2^, Angélica Gallego-Narbón^1^, Alejandro Alonso^1^, & Virginia Valcárcel*^1,3^

**Supplementary Note 1. Conceptual framework and examples of the types of erroneous records.**

The records of the TaxRev database are categorized according to the taxonomic uncertainty of the original identification and the type of taxonomic validation procedure. To carry out this categorization, we first compared the original identification of each record with the revised species identification by V. Valcárcel after the morphological revision of the specimen. Then, we assigned each record to one of the five categories of taxonomic uncertainty (Correct identification, Not identified, Misidentification, Soft taxonomic change, and Hard taxonomic change).

The conceptual framework for the delimiting the categories of taxonomic uncertainty is as follows.

1. Records without taxonomic uncertainty (**Correct identification**). This category identifies records without taxonomic uncertainty as: (i) the original identification of the specimen was correct, and (ii) the scientific name and the taxonomic rank are also correct and updated.
2. Records with taxonomic uncertainty
   1. The source of taxonomic uncertainty arises from the identification task, and it is attributed solely to: (i) the knowledge and/or experience of the identifier in the study group, and/or (ii) the accuracy of the tools used for the identification. These categories identify records with correct names and taxonomic ranks according to the updated taxonomy. Therefore, the uncertainty in these cases cannot be attributed to nomenclatural or taxonomic changes.
      1. **Not identified**: This category identifies records that do not have an original specie-level identification. These records have the maximum possible taxonomic uncertainty (could be any taxa in the genus), but do not produce cascade errors, limiting their use to addressing questions at the species level. Taxonomic validation of these records requires revision of the specimen, either directly or through the photographic documentation if available and useful for this purpose (“hard taxonomic uncertainty” in Fig. 1).
      2. **Misidentifications**: This category identifies records where the original identification of the specimen was wrong. These records have high taxonomic uncertainty and generate cascade errors. Taxonomic validation of these records requires revision of the specimen, either directly or through the photographic documentation if available and useful for this purpose (“hard taxonomic uncertainty” in Fig. 1).
   2. The source of taxonomic uncertainty is attributed to nomenclatural and/or taxonomic changes, and it is reflected in the use of wrong names and/or taxonomic ranks due to outdated taxonomy or nomenclatural confusion.
      1. **Soft taxonomic changes**. This category identifies records with correct identifications but wrong names with or without different taxonomic ranks, which may or may not be associated with a change in the delimitation of the taxa (mergers, splits with spatial segregation, and new taxa). For instance, the records of *H. iberica* originally identified as *H. maderensis* subsp. *iberica* are an example of correct identification with wrong name and different taxa rank but same taxa delimitation. An example of correct identification with wrong name, different taxa rank, and different taxa delimitation are the records of *H. helix* originally identified as *H. helix* subsp. *rhizomatifera.* In this case, the different taxa delimitation is due to the merging of subsp. *rhizomatifera* with subsp. *helix*. In both cases (same taxa delimitation and different taxa delimitation due to merging), the taxonomic validation can be done by following an updated taxonomy and applying a synonym filter. An example where the different taxa delimitation is due to taxa splitting with spatial segregation is the case of the *H. iberica* records originally identified as *H. maderensis*. In this case, the different taxa delimitation is due to the splitting of *H. maderensis* into two new disjunct taxa, one in Madeira and one in the Iberian Peninsula. In this case, the taxonomic validation can be done by following an updated taxonomy and applying a filter combining synonyms and geographical locations. Finally, another possibility of soft taxonomic changes, not represented in our study case, is the discovery of a new species (addition of new taxa). The records included in this category have low taxonomic uncertainty and they can all be validated by data handling since the identification is correct and there is no need to revise the specimen (“soft taxonomic uncertainty” in Fig. 1).
      2. **Hard taxonomic changes**. This category identifies records with wrong names due to taxa splitting resulting in new taxa with no clear geographical segregation (we have no example of this in our study case) or with geographical segregation but nomenclatural confusion (“sensu” in taxonomy, see below). For instance, an example of the latter are the records of *H. iberica* that were originally identified under the epithet (species, subspecies, or variety) “*canariensis*”. On the one hand, these records could be correct identifications of specimens that were incorrectly named. However, these incorrect names cannot be attributed to outdated taxonomy (and therefore cannot be easily validated as 2.2.1), but because of the long-standing nomenclatural confusion of "*canariensis*", which was mistakenly interpreted in a broad sense to include what we now recognise as *H. iberica* and *H. hibernica*. Note that this is not a taxonomic change as the delimitation of *H. canariensis* at any of the taxonomic rank ever recognised has never included *H. iberica* or *H. hibernica*, this is called “sensu” in taxonomy. On the other hand, it is also equally likely that the record was misidentified. That is, the identifier used the correct interpretation of the taxa “*canariensis*” but failed to identify the specimen. In both cases, the taxonomic validation of the specimen requires in-depth knowledge of the taxonomy of the study group and the revision of the specimen, as there is no other way to be sure whether the original source of uncertainty was due to misidentification or nomenclatural confusion (“hard taxonomic uncertainty” in Fig. 1).

**Supplementary Note 2. Geo-referencing and details on the compilation of MixOcc database**

In order to compile the MixOcc database, we first georeferenced the records from **TaxRev**. To do this, we first **transformed** all the original coordinates provided in the records (except those with low precision) to the **WGS84** coordinate reference system. For the remaining records (no original coordinates or low precision), we selected those with detailed location descriptions and used GeoLocate to perform a ***de novo* georeferencing**. Whenever GeoLocate returned more than one option for a given record, we used GoogleMaps to select the most likely location, i.e. the one with the highest probability of the species occurring (forested, riverine or rocky sites). We also used other site descriptors (e.g. distance in km between sites, distance to geographic landmarks) and habitat fields (e.g. on birch forest), and double-checked with Google Earth and Google Street View whether the appearance of the landscape matched a suitable habitat for ivies (forested, riverine or rocky) and the information provided in the habitat description of the record. If none of the most likely locations suggested by GeoLocate were suitable for ivies or matched the habitat description, we discarded the record for georeferencing. We used the same procedure to double check all coordinates provided by GeoLocate, as well as records for which GeoLocate was unable to recover a location.

Each record was assigned a **geographical uncertainty**. For coordinates taken directly from GPS, we used 30m for data collected after 2001, or 70m for older data. For records with original MGR coordinates we applied an uncertainty of 7071.07m for 10,000m grids, 707.1m for 1,000m grids, 70.71m for 100m grids or 7.07m for 10m grids. We did not record the uncertainty provided by GeoLocate while geo-referencing. Because of this, for these records -those *de novo* geo-referenced using Geo-Locate and Google tools-, we decided to be conservative and apply an uncertainty of 10,000m. For records obtained from online repositories, we used the original geographic uncertainty provided. In the case of GBIF records and data obtained from the Azorean Biodiversity Portal (Borges et al., 2010; available in GBIF as "atlantis"), we extracted the geographic uncertainty for each record, as recorded in the "coordinateUncertaintyInMeters" field. For the records obtained from the Biodiversity Data Bank of the Canary Islands (www.biodiversidadcanarias.es), we used 353,55m as the geographic uncertainty, since the grid was 500m.

**DOI references for the original GBIF downloads are:**

*Hedera helix* L.: https://doi.org/10.15468/dl.4h5j94 GBIF.org (18 April 2020) GBIF Occurrence Download https://doi.org/10.15468/dl.4h5j94

*Hedera hibernica* (G.Kirchn.) Bean: https://doi.org/10.15468/dl.rgjk0o GBIF.org (21 February 2019) GBIF Occurrence Download https://doi.org/10.15468/dl.rgjk0o

*Hedera* L.: https://doi.org/10.15468/dl.xhfblr GBIF.org (19 July 2018) GBIF Occurrence Download https://doi.org/10.15468/dl.xhfblr
