# Supplementary material for: High rate of species misidentification reduces the taxonomic certainty of European biodiversity databases of ivies (Hedera L.)
Source: Sci Rep. 2024 Feb 28;14:4876. doi: 10.1038/s41598-024-54735-0 (PMC10902322; doi:10.1038/s41598-024-54735-0)
Supplement: Supplementary file 2 — Supplementary Tables. [file 41598_2024_54735_MOESM2_ESM.docx]

**High rate of species misidentification reduces the taxonomic certainty of European biodiversity databases of ivies (*Hedera* L.)**

Marina Coca-de-la-Iglesia^1,2^, Angélica Gallego-Narbón^1^, Alejandro Alonso^1^, & Virginia Valcárcel*^1,3^

**Table S1.** List of herbaria reviewed. Abbreviations as in the *Index Herbariorum* website (https://sweetgum.nybg.org/science/ih/).

| **Abbreviation** | **Institution name** | **Country, City** |
| --- | --- | --- |
| ARAN | Aranzadi Zientzia Elkartea/Sociedad de Ciencias Aranzadi | Spain. San Sebastián |
| BC | Institut Botànic de Barcelona | Spain. Barcelona |
| BCN | University of Barcelona | Spain. Barcelona |
| BG | University of Bergen | Norway. Bergen |
| BM | The Natural History Museum | U.K. England. London |
| BP | Hungarian Natural History Museum | Hungary. Budapest |
| BR | Meise Botanic Garden | Belgium. Meise |
| BREM | Übersee-Museum Bremen | Germany. Bremen |
| COI | Herbarium of the University of Coimbra | Portugal. Coimbra |
| E | Royal Botanic Garden Edinburgh | U.K. Scotland. Edinburgh |
| FI | Natural History Museum | Italy. Firenze |
| G | Conservatoire et Jardin botaniques de la Ville de Genève | Switzerland. Genève |
| GB | University of Gothenburg | Sweden. Gothenburg |
| GDAC | Universidad de Granada | Spain. Granada |
| HBG | University of Hamburg | Germany. Hamburg |
| HUB | Hacettepe University | Turkey. Ankara |
| JACA | Instituto Pirenaico de Ecología, C.S.I.C. | Spain. Jaca |
| K | Royal Botanic Gardens | U.K. England. Kew. |
| LIV | World Museum, National Museums Liverpool | U.K. England. Liverpool |
| LD | Lund University | Sweden. Lund |
| LOU | AGACAL. Xunta de Galicia | Spain. Pontevedra |
| M | Staatliche Naturwissenschaftliche Sammlungen Bayerns (SNSB) | Germany. München |
| MA | Real Jardín Botánico | Spain. Madrid |
| MGC | Universidad de Málaga | Spain. Málaga |
| MANCH | University of Manchester | U.K. England. Manchester |
| MSB | Ludwig-Maximilians-Universität | Germany. München |
| NMW | National Museum Wales | U.K. Wales. Cardiff |
| O | University of Oslo (O) | Norway. Oslo |
| P | Muséum National d'Histoire Naturelle | France. Paris |
| RNG | University of Reading | U.K. England. Reading |
| S | Swedish Museum of Natural History | Sweden. Stockholm |
| SALA | Universidad de Salamanca | Spain. Salamanca |
| SALAF | Universidad de Salamanca | Spain. Salamanca |
| SANT | Universidad de Santiago de Compostela | Spain. Galicia. Santiago de Compostela |
| SEV | Universidad de Sevilla | Spain. Sevilla |
| VAL | Universitat de València | Spain. València |
| WAG | Naturalis Biodiversity Center | The Netherlands. Leiden |
| WU | Universität Wien | Austria. Wien |
| Z | Herbarium of the University of Zürich | Switzerland. Zürich |
| ZT | Herbarium der Eidgenössische Technische Hochschule Zürich | Switzerland. Zürich |

­­**Table S2.** Summary of data per species for each database. For the TaxRev database, which contains only records of morphologically reviewed specimens, we provide the number of records (populations or localities) and the number of individuals. For the MixOcc database, which contains records with spatial coordinate information, we give the total number of records with the number of records per database (TaxRev and online data sources) in parenthesis.

|  | TaxRev  N records (N individuals) | MixOcc  N records (Rev + Online) |
| --- | --- | --- |
| *Hedera azorica* | 40(91) | 599(3:596) |
| *H. canariensis* | 65(154) | 443(10:433) |
| *H. helix* | 737(1198) | 1835(494:1341) |
| *H. hibernica* | 309(576) | 263 |
| *H. iberica* | 89(180) | 77 |
| *H. maderensis* | 40(77) | 35(33:2) |

**Table S3.** Summary of records per country and per database. For the MixOcc database, the number of records obtained from the TaxRev database and the number of records obtained from online databases are given. Asterisks indicate countries with high taxonomic uncertainty (more than one ivy species in close proximity)

|  | TaxRev | MixOcc |  |  |  | TaxRev | MixOcc |  |
| --- | --- | --- | --- | --- | --- | --- | --- | --- |
| Country |  | TaxRev | Online |  | Country |  | TaxRev | Online |
| Åland Islands | 0 | 0 | 1 |  | Lithuania | 0 | 0 | 7 |
| Albania | 1 | 1 | 2 |  | Luxembourg | 1 | 1 | 3 |
| Andorra | 2 | 2 | 0 |  | Malta | 1 | 1 | 0 |
| Armenia | 4 | 0 | 0 |  | Moldova | 1 | 1 | 2 |
| Austria | 10 | 5 | 38 |  | Montenegro | 0 | 0 | 2 |
| Belarus | 0 | 0 | 3 |  | Netherlands | 3 | 0 | 70 |
| Belgium | 3 | 3 | 46 |  | North Macedonia | 0 | 0 | 3 |
| Bosnia & Herzegovina | 2 | 1 | 1 |  | Norway | 31 | 20 | 43 |
| Bulgaria | 3 | 1 | 8 |  | Palestinian Territories | 2 | 1 | 3 |
| Croatia | 3 | 1 | 13 |  | Poland | 8 | 4 | 122 |
| Cyprus | 2 | 1 | 0 |  | *Portugal | 150 | 88 | 0 |
| Czechia | 2 | 1 | 27 |  | Romania | 3 | 3 | 7 |
| Denmark | 4 | 2 | 74 |  | Russia | 8 | 2 | 16 |
| Estonia | 21 | 9 | 1 |  | San Marino | 0 | 0 | 1 |
| Finland | 0 | 0 | 2 |  | Serbia | 1 | 1 | 4 |
| *France | 39 | 25 | 0 |  | Slovakia | 4 | 2 | 6 |
| Georgia | 3 | 2 | 0 |  | Slovenia | 0 | 0 | 25 |
| Germany | 59 | 39 | 403 |  | *Spain | 606 | 474 | 0 |
| Greece | 38 | 24 | 39 |  | Sweden | 14 | 8 | 129 |
| Hungary | 3 | 1 | 7 |  | Switzerland | 15 | 11 | 21 |
| *Ireland | 8 | 5 | 0 |  | Syrian Arab Republic | 0 | 0 | 0 |
| Israel | 0 | 0 | 8 |  | Turkey | 22 | 15 | 0 |
| Italy | 53 | 47 | 185 |  | Ukraine | 11 | 7 | 18 |
| Lebanon | 2 | 1 | 1 |  | *United Kingdom | 103 | 70 | 0 |
| Libya | 1 | 0 | 0 |  |  |  |  |  |

**Table S4.** Results of the GLM modeling the effects of (i) species and (ii) the expected taxonomic uncertainty per region on the variation in species identification errors in the morphologically revised database (TaxRev) after removing records originally identified by *Hedera* taxonomists were removed (N = 1011 records). Factors with significant *p*-values are shown in bold. ****: p ≤ 0.0001, ***: p ≤ 0.001, **: p ≤ 0.01, *: p ≤ 0.05, m.s., 0.05>p≤0.1; n.s.: p > 0.1

|  | Estimate | Std. Error | Error z value |
| --- | --- | --- | --- |
| *H. azorica* | 0.02487 | 0.34715 | 0.072^n.s.^ |
| *H. canariensis* | -0.03712 | 0.43810 | -0.085^n.s.^ |
| ***H. helix*** | -2.33959 | 0.36141 | -6.473**** |
| *H.* *hibernica* | 0.56541 | 0.42474 | 1.331^n.s.^ |
| ***H. iberica*** | 2.43854 | 1.07405 | 2.270* |
| *H. maderensis* | 0.60474 | 0.60722 | 0.996^n.s.^ |
| **Expected taxonomic uncertainty per region (1, 2 or 3 spp.**  **)** | 0.59417 | 0.10305 | 5.766**** |

**Table S5.** Summary of species identification errors within each of the five regions of expected high taxonomic uncertainty (i.e., regions with 2 or 3 spp. sharing range boundaries) as obtained from the morphologically revised database (TaxRev) after removing records originally identified by *Hedera* taxonomists were removed (N = 556 records). Results from the chi-squared tests with significant values are in bold. ****: p ≤ 0.0001, ***: p ≤ 0.001, **: p ≤ 0.01, *: p ≤ 0.05, n.s.: p > 0.05. Mis: incorrect identifications attributed to misidentifications; NoIden: no original identification at the species level. Hard: incorrect identifications attributed to hard taxonomic changes (changes that cannot be validated by standard taxonomic validation procedures). Soft: incorrect identifications attributed to soft taxonomic changes (changes that can be validated by standard taxonomic validation procedures).

|  | N | Correct:Incorrect identifications | Correct  identifications | Incorrect  identifications | Incorrect  identifications | | | | |
| --- | --- | --- | --- | --- | --- | --- | --- | --- | --- |
|  |  |  |  |  | Mis | NoIden | Hard | Soft | |
| France (2 spp., *H. helix* and *H. hibernica*) | 33 | 21:12*^n.s^* | 21 | 12 | 1 | 8 | 0 | 3 |  |
|  |  |  | 64% | 36% | 3% | 24% | 0% | 9% |  |
| Ireland  (2 spp., *H. helix* and *H. hibernica*) | 8 | 0:8^NA^ | 0 | 8 | 7 | 1 | 0 | 0 |  |
|  |  |  | 0% | 100% | 88% | 13% | 0% | 0% |  |
| **mainland Portugal**  **(2 spp., *H. hibernica* and *H. iberica*)** | **52** | **8:44*^****^*** | 8 | 44 | 4 | 24 | 16 | 0 |  |
|  |  |  | 15% | 85% | 8% | 46% | 31% | 0% |  |
| **mainland Spain**  **(3 spp., *H. helix, H. hibernica* and *H. iberica*)** | **363** | **135:228*^****^*** | 135 | 228 | 99 | 103 | 1 | 25 |  |
|  |  |  | 37% | 63% | 27% | 28% | 0% | 7% |  |
| UK  (2 spp., *H. helix* and *H. hibernica*) | 100 | 49:51*^n.s.^* | 49 | 51 | 38 | 8 | 0 | 5 |  |
|  |  |  | 49% | 51% | 38% | 8% | 0% | 5% |  |
